# Supplementary material for: Effects of Destruxin A on Silkworm’s Immunophilins
Source: Toxins (Basel). 2019 Jun 18;11(6):349. doi: 10.3390/toxins11060349 (PMC6628623; doi:10.3390/toxins11060349)
Supplement: Supplementary file 1 [file toxins-11-00349-s001.pdf]

Supplementary Materials: Effects of Destruxin A on Silkworm's Immunophilins

Jingjing Wang, Qunfang Weng and Qiongbo Hu *

Commented [M1]: Please carefully check the accuracy of names. Changes will not be possible after proofreading.

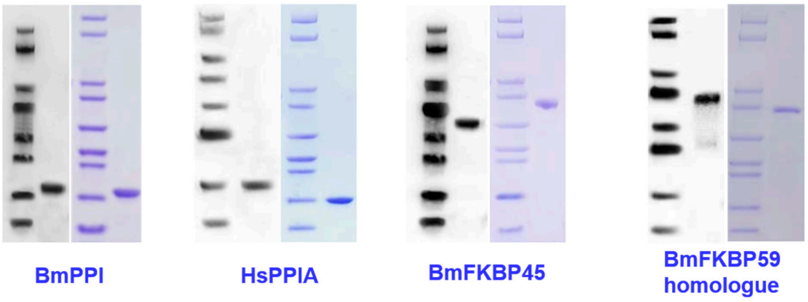

Figure S1. All proteins in this paper were successfully expressed and purify in *E coli*. Left part of each pictures were western blot verification of expressing, right part were verification of purification. Maker of each picture from top to bottom are 160, 120, 70, 50, 40, 35, 20, 10 kD.
